# Integrating EQUIP competency-based training into a university curriculum: a qualitative inquiry with students and faculty at Makerere University in Uganda

**DOI:** 10.3389/feduc.2023.1290630

**Published:** 2024-01-05

**Authors:** Morris Ndeezi, Gloria A. Pedersen, Benjamin Alipanga, Ibrahim Luberenga, Brandon A. Kohrt, Roscoe Kasujja

**Affiliations:** 1 Department of Mental Health, Makerere University, Kampala, Uganda; 2 Center for Global Mental Health Equity, Department of Psychiatry, George Washington University, Washington, DC, United States; 3 Department of Mental helath, Makerere University, Kampala, Uganda; 4 Michigan State University, East Lansing, MI, United States

**Keywords:** competency-based training, role-play, feedback, competence, clinical psychology, EQUIP

## Abstract

**Introduction:**

Competency-based training has gained prominence in clinical psychology education, emphasizing practical skills acquisition. The EQUIP competency-based approach, recognized for its effectiveness in in-service training, raises questions about its feasibility and utility in pre-service education.

**Methods:**

Faculty and supervisors were trained in and applied EQUIP competency-based assessment and techniques with current graduate students. A cohort comprising 15 graduate students, 12 faculty members, and supervisors from Butabika National Referral Mental Health and Teaching Hospital participated in focus group discussion and key informant interviews. Qualitative data was collected from 1st August 2022 to 4th August 2022. Thematic analysis identified three central themes.

**Findings:**

The first theme reveals participants’ recognition of EQUIP’s feasibility in standardizing competence levels and addressing curriculum gaps. An extended training period, approximately 15 h, was identified as crucial to enhance educators’ and supervisors’ confidence in implementing the approach. The second theme emphasizes the pivotal role of role-play in competency-based training, transforming initial apprehension into constructive learning. Lastly, feedback emerged as a crucial component, with participants highlighting its role in fostering self-assuredness and refining skills.

**Conclusion:**

The study emphasizes the importance of robust training in competency-based methodologies. The EQUIP approach’s potential in clinical psychology education is evidenced by its alignment with research outcomes. Beyond this, the study advocates for longitudinal research to assess sustained engagement with EQUIP resources and their long-term impact. This research not only advances the discourse on competency-based training but also sets the stage for continuous improvements in clinical psychology education.

## Introduction

In the ever-evolving area of clinical psychology education, where mental health concerns in Uganda require immediate attention, Competency-Based Education through the EQUIP approach has emerged as a new paradigm. The need for creative training methods that not only meet but also exceed industry standards is growing as the mental health landscape shifts. In Uganda, 13 % of the country’s disease burden is caused by mental health issues (Kigozi et al., 2010; Wakida et al., 2019). A recent report indicates that over 11 million people in Uganda have mental health problems (The New Vision, 2022). Uganda faces the same problems as the majority of black African nations of the Sub-Saharan, including poverty and underdevelopment, child abuse and neglect, trauma brought on by various types of crime and abuse of human dignity, kidnapping, and hijacking, rising stress-inducing lifestyles, westernization and globalization, the HIV/AIDS pandemic, and various cancers (Kiima et al., 2004; Mayeya et al., 2004; Ndyanabangi et al., 2009; Opio et al., 2022). The adverse emotional consequences of such stressors need a well-trained mental health workforce who can utilize suitable psychotherapy techniques that are safe and effective in treating clients, and that will be appealing to them (Madu, 2016). However, in Uganda, there is still a significant gap between those who require mental health services and those who can get it, with an estimated treatment gap as large as 85% (Lund et al., 2012; Shidhaye et al., 2015).

To better fulfill the ongoing and changing mental health requirements of communities, there have been an increasing number of requests in recent years for the education of mental health professionals to be changed using a competency-based approach (Kiguli-Malwadde et al., 2014; Mills et al., 2020). Particularly in the field of clinical psychology, competency-based education is receiving more attention as a potential solution to some of the problems associated with mental health training (Hall et al., 2014; Stevens et al., 2017; Mills et al., 2020). It has also been traditionally used in the medical field. It takes an outcomes-based approach, wherein the required competencies form the basis for establishing training objectives, planning training activities, and carrying out assessments to ensure competent professionals (Kaslow, 2004; Kaslow et al., 2004). Competent mental health providers are more likely to provide clients with psychological therapies that are efficient and secure (McHugh and Barlow, 2010). Although many programs have followed a competency-based model, each program is unique according to the tools and strategies used, and therefore there is a lack of consistency in terms of how competency is measured and assessed, including among clinical psychology students training in Uganda. For this reason, clinical psychology programs in Uganda require consistent use of standardized tools and guidance to competency-based training to ensure a minimum standard of competency for safe and effective mental health intervention.

In March 2022, the World Health Organization (WHO) and The United Nations Children’s Fund (UNICEF) developed the Ensuring Quality in Psychological Support (EQUIP) standard method (Kohrt et al., 2020). The EQUIP platform is meant to support trainers and supervisors in using a competency-based approach to ultimately ensure safe and effective mental health intervention (Kohrt et al., 2020). As a digital tool, which contains both online and offline formats, it offers resources that support the training and supervision of mental healthcare of adults, children and adolescents, as well as groups (Kohrt et al., 2015; Jordans et al., 2021b; Pedersen et al., 2021b). The EQUIP platform generously provides an array of competency assessment tools. First and foremost, it addresses the foundational competencies crucial for delivering effective mental health care to adults. (e.g., communication, empathy, promoting hope) [ENhancing Assessment of Common Therapeutic factors (ENACT)] (Kohrt et al., 2015; Singla et al., 2017; Pedersen et al., 2020), with children,– Assessment of Competencies Tool (WEACT) (Jordans et al., 2021a) and adolescents, and core skills for facilitating groups Group facilitation Assessment of Competencies Tool (GroupACT) (Pedersen et al., 2021a). The platform also includes treatment package competencies for WHO interventions guides such as Problem Management Plus (PM+) and the Thinking Healthy Programme (THP), as well as condition-specific techniques for diverse therapy classes including cognitive, problem-solving, interpersonal, trauma-related, motivational enhancement, and stress management techniques (Pedersen et al., 2020, 2021a). The EQUIP platform provides direction on how to perform role plays for competency-based training, which includes competency-based feedback into the training, and a feature that tracks data and enables data to be visualized which enables changes in competency over time to be followed.

The digital platform makes it simple to assess competencies during and after training and supervision programs, allowing trainers and supervisors to immediately evaluate learners (e.g., clinical psychology students) and to track their competency progress over time. On the platform, feedback is automatically displayed to highlight learners’ achievements and any potentially harmful behaviors that require extra teaching prior to working with patients in the real world. The use of EQUIP platform has been reported to be effective in boosting mastery of foundational helping skills as well as reduce harmful behaviors (Jordans et al., 2022), when compared to other non-competence-based programs.

The EQUIP competency-based approach’s viability and acceptability have been investigated in a number of in-service training scenarios. Studies by Mathai et al. (2023) and Jordans et al. (2022), among others, have shown the viability and acceptance of the technique for teaching non-specialists, mental health professionals, and group-based therapies. These research showed that the technique improved skills and competence among various target groups and showed efficacy in various situations, and as such, was received favorably. Furthermore, Jordans et al. (2021b) examined the viability and acceptability of the EQUIP method in the context of training and oversight for Psychological First Aid (PM+) practitioners. Their findings provided important new information on the viability and acceptability of the strategy in this particular domain. A research by Jordans et al. (2021a,b) on the viability of evaluating competences using the EQUIP technique was carried out in Zambia, and the results were helpful for understanding how to assess and rate competencies in a specific environment. However, it is important to note that there is a gap in research regarding the feasibility and acceptability of the EQUIP competency-based approach in pre-service training programs, such as those conducted in university settings. The department of Mental Health and Community Psychology at Makerere University offers a Master of Science degree in Clinical Psychology. The courses are taught through interactive learning approaches including tutorials, fieldwork, case studies, small group and class discussions, and self-study. Students on practical and internships are trained through actual work with patients under the supervision of a placement site supervisor and a university supervisor. Students attend weekly class consultations and supervision with the course coordinator to discuss their practicum or internship experiences. Formative and summative evaluations are based on the learning strategies used, such as case studies based on patients, class presentations on predetermined subjects, independent study, and prescribed readings. For certification, a student must successfully complete all courses, including the final exam, with a minimum cumulative grade of 60% obtained through the various evaluation methods mentioned above. However, there is inconsistency in the methods of training, assessment and supervision of the targeted psychology competencies. The choice of training, assessment and supervision is not regulated and is left up to the discretion of the individual course lecturer. As a result, a trainee might do well on all knowledge-based exams but badly on skill-based evaluations and still successfully finish the master’s degree course since the 60% minimum criterion does not distinguish between knowledge and skill-based evaluations. As such, the clinical psychology program at the Makerere University seems to be an ideal place to incorporate a competency-based approach using the EQUIP tools and resources to standardize assessments and feedback.

According to Alipanga and Kohrt (2022), formal training institutions are best placed to ensure the sustainability of training and competency maintenance for effective mental health care. Moreover, if specialist pre-service programs integrate resources such as EQUIP, this prepares their graduates to conduct competency-based approaches in their future training of both specialists and non-specialists, and thus creating multiple effect. Although EQUIP competency-based approaches have been integrated in some parts of Africa (Pedersen et al., 2021a; Mathai et al., 2023), it has not yet been systematically evaluated and integrated into the pre-service training of mental health professionals. In response to the need for mental health service providers with skills to manage the unique problems of LMIC like Uganda, Alipanga and Kohrt (2022) proposed even steps to develop, implement, and evaluate a competency-based curriculum in pre-service training. Competency-based training focuses on specific and predetermined skills which are taught, assessed and supervised during training. Accordingly, a 3-day training on how to use EQUIP was held among faculty and clinical supervisors from Makerere University and Butabika Hospital. Here, they learned how to rate competency assessment tools, how to perform in competency-based role plays, how to provide competency-based feedback, and held discussions on best ways to assess and provide feedback with students in classroom settings. A sample training agenda can be found in the supplement. This training was directly followed by a half-day of brief, 10-min role play based competency assessments and tailored feedback sessions with current clinical psychology students at Makerere University.

Therefore, this study aimed to investigate the experiences of faculty and clinical supervisors implementing an EQUIP competency-based approach, and experiences of clinical psychology students participating in competency-based role plays assessments and feedback sessions. We explored areas of feasibility and utility of incorporating an EQUIP competency-based approach within the clinical psychology curriculum for standardizing the training, assessment and supervision of pre-service students at Makerere University.

## Materials and methods

Focus groups and in-depth interviews were used to collect data from 27 participants on the feasibility and utility of using the EQUIP protocol. The participants were purposively selected and comprised faculty and pre-service students from the Department of Mental Health and Community Psychology at Makerere University, and mental health practitioners from Butabika National Referral Mental Health and Teaching Hospital who are involved in supervising the mental health service competencies of the students.

### Procedures

Faculty at the Makerere University and Butabika Hospital in Uganda had a 3 days training about the implementation of a competency-based approaches using tools and resources provided on the WHO/UNICEF EQUIP digital platform. The faculty at Makerere University applied to learn by performing standardized role-play assessments using the ENACT and feedback piloted the use of the EQUIP protocol in training, assessing and supervising pre-service students of clinical psychology at the university in order to establish the feasibility and utility of the protocol in a formal training institution. In-depth interviews were held with the faculty and student supervisors from Butabika National Mental Health Referral and Teaching Hospital on the days of the training and participated in a FGD at the end after the assessment. The students held two FGDs after participating in role-plays and were given feedback about the role-plays.

All participants participated voluntarily and gave their verbal and written consent after thorough information about the study in both oral and written form. Participants had time for reflection and were able to ask any questions to the researchers before the written consent was given. Confidentiality was emphasized to all participants, along with the rules of the interview, in the beginning of each interview. They were familiar with the possibility of ending the interview with no following consequences, and only to tell and share what they found acceptable. Participants were pseudo-anonymized and the initials presented in the text are fictive. IRB approval was obtained from the Institutional Review Board (IRB) of Makerere University School of Health Sciences Research Ethics Committee (MAKSHSREC-2022–285).

### Participants

The qualitative study enlisted the participation of fifteen graduate students pursuing clinical psychology, along with twelve faculty members from Makerere University and supervisors from Butabika National Referral Mental Health and Teaching Hospital. A heterogeneous purposive sampling strategy was meticulously employed to ensure the inclusion of the intended participants. The eligible participants encompassed faculty members from the Department of Mental Health and Community Psychology, students pursuing Masters of Clinical Psychology within the same department, and mental health practitioners responsible for supervising clinical psychology students during their practicum at Butabika National Referral Mental Health and Teaching Hospital. To equip the faculty and supervisors with the necessary insights, a comprehensive three-day training was conducted. Immediately following the training, they effectively translated their acquired knowledge into practice by evaluating the foundational helping competencies of the clinical psychology students through standardized role-playing scenarios. Every student engaged in role-plays subsequently partook in focused group discussions (FGDs). The involvement of faculty and internship supervisors extended to both in-depth interviews and FGDs. Notably, the faculty and supervisors who underwent the training boasted extensive experience in both training and clinical supervision concerning the students. The focus group discussions comprised five to twelve participants each, categorically assembled. Within this structure, one FGD was convened for the participants of the training, specifically the faculty and supervisors. In addition, two separate FGDs were orchestrated for the students.

### Data collection method

The interviews encompassed both students and supervisors and spanned a duration of 20–45 min. This interview process was divided into two distinct sets. The initial set was conducted after 5 h of training on the first day of the program. The second set occurred during focused group discussions (FGDs) after a comprehensive 15-h training period, which concluded with role-play assessments and feedback on the final day. These interviews were meticulously recorded, strictly following the participants’ granted permissions. Participants were reassured of the utmost confidentiality concerning their workplaces and roles. They were explicitly informed that the information shared would solely serve the study’s purpose and that their identities, as well as their workplaces, would remain entirely anonymous in any subsequent reports. Post-interviews, the recordings underwent transcription and were stored securely as computer files. The researchers took conscientious measures to ensure the research report’s final version and the interview recordings were safeguarded under lock and key. This approach guarantees the information’s privacy and preserves the integrity of the study’s findings.

### Data analysis

We applied thematic analysis, a method recognized for its effectiveness in uncovering patterns and themes within qualitative data. Thematic analysis was chosen due to its suitability in exploring participants’ perceptions and experiences (Fugard and Potts, 2015). This approach involves several key steps, each contributing to a comprehensive understanding of the collected data. The initial step involved familiarization with the data, wherein all interview recordings and transcriptions were repeatedly reviewed to establish a sense of immersion. Subsequently, initial codes were generated, systematically identifying meaningful phrases, sentences, or paragraphs that encapsulated the data’s essential aspects. Following this, codes were collated into potential themes, considering their relevance and cohesiveness with the research objectives. The themes underwent a meticulous review process, refining their definitions and boundaries to ensure clarity and accuracy. These refined themes were then organized into a coherent structure, capturing the essence of participants’ insights while maintaining a logical flow. This stage aimed to construct a narrative that aligns with the research’s objectives. Throughout this process, a rigorous approach was taken to maintain the research’s integrity. The analysis was both iterative and reflexive, involving multiple researchers in reviewing and validating the identified themes. This ensured that the findings genuinely represented the participants’ perspectives. Thematic analysis facilitated the identification of recurring patterns, enabling a nuanced exploration of the EQUIP approach’s feasibility and effectiveness. This methodological approach enhances the study’s robustness, enabling a rich understanding of participants’ viewpoints and experiences.

## Results

Three themes emerged during the analysis: (a) The feasibility and usefulness of the EQUIP resources in training, assessing and supervision of competency, (b) Experience of using role plays and integrating them in the training and supervision of the students (c) The benefits and the challenges of giving and receiving feedback.

### Theme A: the feasibility and usefulness of the EQUIP resources in training, assessing and supervising of competency

Participants’ provided insights into their perception of the feasibility and usefulness of the EQUIP resources for training, assessing, and supervising competency. This theme explored their views on the assessment approach, use of role plays, training structure, knowledge of EQUIP, and the use of the ENACT tool. The results under this theme is presented progressively from the interviews on Day one and the FDGs on the last day.

After a comprehensive five-hour training session on the first day, participants were questioned about their comprehension of the EQUIP approach. Faculty members and supervisors demonstrated a clear understanding of EQUIP’s essence, with one practicum supervisor succinctly summarizing it as,

“EQUIP is an approach to assessing, training, and supervising individuals in the mental health field. It focuses on assessing skills and competence, particularly in delivering interventions in a helping profession.” (Onsite Supervisor)

The participants were also prompted to share their expectations and what they hoped to learn in the upcoming training days. Notably, the need for a more profound understanding of the assessment procedure under EQUIP emerged. A participant articulated this need, stating:

I want to look at the detail of this assessment. Now it looks like an introduction to me. So possibly now we are getting into detail in the coming days of the training. (Onsite Supervisor)

Additionally, participants expressed the desire for further clarification on using the assessment tools, particularly regarding the various levels (Level 1 through Level 4) and the corresponding remediation strategies based on the attained level. One practicum supervisor voiced their query:

“I would like to learn more about the different levels of ENACT and how they work. Are there specific interventions for each level? If someone moves from level one to level three, should they go back to the basic competencies?” (Onsite Supervisor)

As the training progressed, after a cumulative 15 h, participants were prompted to share their perceptions about the feasibility and utility of the competency-based approach, as well as their overall training experience. A faculty member expressed optimism about the EQUIP approach, viewing it as an avenue to enhance the current training of clinical psychologists. He explained that:

“…yeah, if we go on with such a system of [competency-based] training it would have been really good. …it will work very much for improvement of training, if it gets to be done like that continuously, it will be very helpful.”(Faculty)

Another faculty member stated that the EQUIP tools specifically ENACT can help in assessing the impact of the trainings conducted:

“We have done training before in our organization and we have been wondering, ‘Did it actually create an impact? How can we assess impact?’ So far, I think the tool that we have gone through, the ENACT tool, can give us that opportunity to strive and actually assess using an evidence-based structured way.” (Faculty)

Regarding the feasibility of using the EQUIP/ENACT tool for training, assessment, and supervision, participants noted that the EQUIP enables the establishment of clear learning objectives and the assessment of learning outcomes as important. A supervisor mentioned:

“The ENACT helps structure training and assess the learning outcomes of various elements. For example, we can train on confidentiality and assess confidentiality skills. We can also train on assessment and determine whether participants have learned.” (Onsite Supervisor)

Additionally, another participant reported that because the ENACT tool is structured, detailed, and user-friendly in nature, it is practical for learners. One participant had this to say:

“The ENACT tool is structured, detailed, and simple to use. It can be applied to students and learners in practice, as it covers relevant basic skills. It serves as an excellent guide.” (Onsite Supervisor)

Overall, after 15 h of training, the participants became more confident in using the tool and appreciated the EQUIP approach to competency-based training, regarding to it as a novel and structured approach to measuring competence that ensures standardized quality across different educational institutions. One participant noted:

“… Actually having a structured format will mean that the students that are being produced from different universities have the same standard. Everyone will know that we have students (trained) at a certain level of competence. So in other words, even if you are from different universities we can know that this person has gone through this competence training and they have passed, which means they have a certain level of skills.” (Faculty)

Another faculty member added that;

“In the past, there was a lack of a structured way to measure students’ competence. With EQUIP, different universities can have the same standard for assessing competence. “We can confidently identify students who have gone through this competence training and have reached a certain level of skills, regardless of their university.” (Faculty)

In summation, participants recognized EQUIP as a feasible and invaluable resource for training, assessing, and supervising competency within the realm of mental health. Their initial need for enhanced training was effectively addressed throughout the program, culminating in a profound grasp of EQUIP’s purpose and advantages. The structured approach of EQUIP, coupled with the efficacy of the ENACT tool, garnered praise for their ability to set precise learning objectives, evaluate learning outcomes, and ensure uniform competence measurement.

### Theme B: experience of using role plays and integrating them in the training and supervision of the students

This theme explores the participants’ experiences with using role-plays and integrating them into the training and supervision of students. Three sub-themes were identified under this theme which comprised of their experiences of using role-plays, the learning derived from them, the structure of role-plays, and their implementation.

### Subtheme B.1: experiences of using role-plays

When asked about their experiences with using role-plays in training, assessment, and supervision of competencies, the participants reported that role-play training facilitated the observation of students’ progress and instilled confidence in their ability to perform tasks in real-life practice. One participant expressed this sentiment:

“When you are training people through role-plays, you can easily assess their improvement. If they struggle to perform a task in a role-play, it implies that they will face difficulties in executing it with actual clients.” (Faculty)

Some of the participants emphasized the importance of role-plays in identifying and replacing unhelpful behaviors with more effective ones, and the use of feedback to help learners improve the identified behaviors. Regarding this, one participant had this to say:

“I found the role-play process highly beneficial. Using the tool, it was easy for us to identify harmful or less helpful behaviors and work on removing them while focusing on strengthening essential skills during feedback.” (Onsite supervisor)

Another participant acknowledged that role-plays enabled students to identify their strengths and weaknesses, serving as a motivating factor for self-improvement. The participant stated:

“Most students were able to recognize their strengths and weaknesses through role-plays. This awareness drives them to improve their performance in subsequent role-plays and assessments. Reflecting on weaknesses helps us monitor progress in various competencies.” (Faculty)

All students endorsed the implementation of role-plays in the assessment and training of competence and believed that it enables actions to be observed and competencies to be evaluated, and therefore determine readiness for work in real life. One student captured this by saying:

“In therapy practice, it is not about documenting what we do but about the supervision process. [Through role plays] supervisors can observe us in action, evaluate our competence, and determine if we are ready. Implementing role-plays would provide a comprehensive assessment of our abilities.” (Student)

However, there were some reservations about the use of role-plays. Some students expressed anxiety during role-plays, feeling self-conscious and under pressure due to the presence and assessment by supervisors. Additionally, the allotted time for role-plays (10 min) was perceived as insufficient. One student shared this experience:

“When told that you only have ten minutes for a session, even if you are well-prepared, anxiety can creep in. You become anxious about managing time and demonstrating everything required within such a limited period.” (Student)

Similarly, another student admitted feeling nervous during role-plays but found the overall experience enlightening, particularly the feedback sessions. They stated:

“I was nervous during the role-play, but the feedback was valuable. It helped me recognize areas for improvement. I appreciate this experience.” (Student)

Additionally, some participants who took part in student assessment reported that anxiety indeed affected the students’ performance, and they suggested that additional support should be provided to help students relax before the sessions. One participant commented about this thus:

“The instructions are clear, but we need to understand that students may feel anxious during assessment sessions. It is important for us to help them calm down and alleviate their anxiety, as this might lead to skipping important elements.” (Faculty)

While most participants agreed on the benefits of role-plays for training purposes, some had reservations about using role-plays as the sole method for evaluating students’ skills. One participant stated:

“Role-plays are excellent for training, but they may not be the most effective approach for evaluating practitioners’ skills. However, if integrated continuously into training, they can be highly beneficial.” (On-site Supervisor)

Another participant echoed similar thoughts and expressed concerns about the applicability of skills learnt through role-plays in real life situations. The participant said:

“Assessing competence through role-plays is crucial during training as it provides insights into individuals’ readiness for real work. However, success in role-plays does not guarantee the same performance in actual practice. Anxiety can negatively affect their performance.” (Onsite Supervisor)

In summary, participants believed role-plays were useful because they enable identification of strengths and weaknesses of learning, and thus enable relevant improvements and confidence building in the learners. They recommended the use of role-plays in training, assessment, and supervision for prospective trainees to promote skill development for effective mental health service.

### Subtheme B.2: learning from the role-plays

The majority of students reported that participating in role-plays provided them with valuable insights into their strengths and weaknesses. One student reported that:

“The role-play exercise was incredibly educational and made us aware of our current baseline skills. It was a great experience because we learned about our strengths and weaknesses from others. Overall, it was a highly valuable experience.” (Student)

Another student expressed a strong support for using role-plays because they enable identification of existing competencies and therefore lead to the consolidation of the competencies. The student said:

“I highly recommend the continuation of role-plays because they help us acknowledge our skills and provide opportunities for improvement. Follow-up sessions would be valuable to ensure mastery and practical application. From my perspective, this exercise is highly recommended.” (Student)

Furthermore, some students expressed the desire to have more role-play session, possibly involving real patients and longer observation and feedback periods in order to master the competencies being learnt better. The following are some remarks by the students:

“We need more role-plays and more practice. It should not be a one-time event. Immersing ourselves in practice and having more role-plays will lead to better results.” (Student)

Another student mentioned;

“I have thoroughly enjoyed the role-play experience, and I hope it can be repeated with more time. It would be ideal to have an entire session where we work with a real patient under the supervision of our mentor until we fully learn. I’m not sure if it’s feasible, but it would be a valuable approach.” (Student)

### Subtheme B.3: structure of the role-plays

Participants appreciated the clarity and adequacy of the instructions provided before the role-plays. They found the instructions informative enough to carry out the role-plays effectively. One participant expressed their opinion:

“The role-play instructions were clear, especially regarding the fact that the interviewer had not met the client before. This prompted the students to ask for the client’s name and reason for their visit. The instructions provided just enough information, particularly for skills like building rapport. I found them to be useful.” (Faculty)

However, some participants felt that the instructions for executing the role-plays were insufficient. For example, one participant noted an issue with the role-play script, where only one name was provided, leading to a weakness in the performance:

“One student asked me for my second name, but the role-play script only provided one name. This created confusion and impacted the overall performance.” (Onsite Supervisor)

Another participant observed that several students did not ask the reason why the client visited their office, attributing this to the instruction in the role-play script. They stated:

“At the beginning, it seemed like students were instructed not to ask the ‘presenting problem’, resulting in two out of three students missing the client’s reason for seeking help. It appeared that they took the instruction about the client being distressed as the reason for the visit.” (Onsite Supervisor)

Many students expressed dissatisfaction with the limited time allotted for the role-plays. They felt that 10 min was insufficient to assess a person’s competence and that it only allowed for the opening phase of a session. The time constraint induced anxiety even before the sessions began. One student shared their experience:

“The most challenging aspect was the time limitation. I agree with my peers that being given only ten minutes for a session with a client presenting their problems is difficult. Even if you are well-prepared, your performance is affected by the limited time.” (Student)

Another student expressed a desire for more time, acknowledging the value of being trained as time managers but also recognizing the need for an extended role-play and feedback process:

“Personally, I found the experience enjoyable, although the time provided was very short. Nonetheless, I took it as an opportunity to practice time management. However, I would appreciate more time for the role-play and feedback process, perhaps an entire session under observation and feedback.” (Student)

### Theme C: the benefits and the challenges of giving feedback

Benefits and challenges associated with the process of giving and receiving feedback are the two sub-themes that emerged from this section. The section describes the participant’s perception of giving feedback to the clinical psychology students after a role play. It also covers the benefits and the challenges of feedback.

### Subtheme C.1: benefit of giving feedback

From the students’ perspective, feedback played a critical role in helping them identify their strengths and weaknesses. Positive feedback was particularly encouraging as it highlighted specific competencies possessed by the students. Additionally, some students embraced negative feedback as an opportunity to address weaknesses and motivate themselves to practice more in order to overcome perceived limitations. One student reflected on this, stating,

“Receiving feedback made me aware of skills I did not know I possessed as an individual. I also appreciated the negative feedback because it revealed areas where I needed further improvement. Overall, the experience was enlightening.” (Student)

Another student echoed similar sentiments, expressing,

“I found the experience (of feedback) to be positive. If we engage in more exercises like these and receive feedback, we can enhance our learning and gain a better understanding of what needs to be done. Sometimes, we may be moving forward without knowing the right direction. Engaging in role-plays and receiving feedback can be immensely beneficial.” (Student)

One student emphasized the transformative impact of receiving feedback on a personal level compared to group feedback in a classroom setting. The student explained that individualized feedback is very impactful and fosters a strong sense of responsibility and the motivation for improvement. One of them stated,

“Having the opportunity to receive feedback personally has been truly impactful. It delves deep within, compelling me to put in the necessary effort. When feedback is delivered personally, it becomes my responsibility to work on it. Thus, I consider it a more valuable experience.” (Student)

The majority of students who received feedback exhibited positive emotional reactions. Students particularly appreciated feedback that focused on their strengths, as it boosted their confidence and enabled them to relate to the facilitator’s comments.

“I observed a student over there receiving positive feedback and I could see her glow, her face glowed with confidence. The feedback is confidence building for her and she was able to relate to what the facilitator was saying.” (Student)

A student stated that feedback help him cope with anxiety of the things he did not do well in the role-play. He stated:

“I was helped to know how to cope with my inadequacies. I should use the word “anxiety” for some of those things that I did not do well.” (Student)

### Subtheme C.2: challenges of giving and receiving feedback

Although most students accepted the feedback provided, the raters noted that some few students exhibited resistance, claiming that it contradicted what they had been taught in class. The raters clarified that this resistance did not indicate any flaw in the students’ prior training. Instead, they reinforce the importance of flexibility and the application of clinical judgment in real-world practice. One onsite supervisor shared their experience, saying,

“During the feedback session, two students repeatedly expressed that their training instructed them otherwise. We did not perceive it as negative, but it seemed like they were resistant to feedback. When students assert that their training conflicts with the feedback I provide while supervising, it can pose challenges.” (Onsite Supervisor)

Some students commented on the same issue as the supervisors, some found some contradictions between what they were originally taught and what was expected of them in the assessment. They believe that having a clear standard for what is appropriate and what is not would be good for them as one of them stated:.

“I think having a clear benchmark of what is appropriate and what is not would have been a better option because even right now we have left the assessment we have been assessed by different people we all got different opinions and therefore we do not know what is right, what is wrong because he has now told me he was told he was laughing at the client and yet he was laughing with the client, Sanyu (not real name) is saying she took three minutes so each of us now has these issues but I think if you guys had come up with a bench marker of uniformity to ensure that everyone must meet this criterion it would have been a better way of assessing than just that way of assessing based on the individual.” (Student)

However, it was observed that some students reacted defensively when receiving negative feedback. One participant acknowledged this phenomenon, stating:

“Conversely, I also observed a defensive reaction from a student when confronted with what he perceived as negative feedback.” (Faculty)

The participants emphasized the importance of considering various factors when providing feedback to students, with a particular emphasis on the need for sensitivity in choosing an appropriate starting point. According to the participants, starting on the negative feedback can lead to defensiveness in students. To address this, one participant suggested beginning with a positive feedback or asking students to share their impressions of the session, which establishes expectations and lays the groundwork for constructive feedback. They explained,

“Starting with negative feedback can activate the defensive gear in students, so it is beneficial to begin on a positive note. If starting positively feels challenging, asking students about their feelings and identifying three positive aspects of the session can provide valuable insights.” (Faculty)

In conclusion, the participants’ insights shed light on the benefits and challenges associated with giving and receiving feedback. Feedback was found to enhance self-awareness, facilitate the identification of strengths and weaknesses, and serve as a source of motivation for improvement. The challenges identified included the importance of sensitivity in delivering feedback, the importance of positive feedback, and uncertainty about the apparent conflict between classroom teachings and real-world application. These findings underline the importance of the dynamics of feedback and its role within the context of competency training, assessment and supervision/feedback.

## Discussion

This study significantly enhances the ongoing discourse pertaining to competency-based education within the clinical psychology domain. The findings robustly affirm the viability and reception of the EQUIP competency-based approach, not only in in-service training but also in pre-service contexts. This addresses a crucial void stemming from the absence of standardized curricula for teaching clinical psychologist in Uganda. Moreover, the alignment of this study with prior research bolsters the credibility of the EQUIP approach and magnifies its transformative potential for reshaping clinical psychology education.

Numerous studies focusing on the feasibility and acceptance of the EQUIP competency-based approach in in-service training consistently highlight its viability and positive reception (Jordans et al., 2021b, 2022; Mathai et al., 2023). These antecedent findings are consonant with this research, demonstrating that the EQUIP approach holds equal relevance and importance for pre-service training and the mentorship of clinical psychology students. Participants in this study acknowledged its potency in establishing uniform competence levels and rectifying the absence of standardized curricula, aligning with the overarching goal of fostering adept professionals. This resonates with extant literature on competency evaluation, which accentuates the pivotal role of real-world application and readiness during assessments (Sheen et al., 2015).

The qualitative findings of this study had a striking resemblance to fundamental topics covered in the brief (~15 h) training. Notably, several concerns raised by participants earlier in the training, such as how to rate the ENACT tool, were addressed through proceeding training sessions that included role play practice, as well as real-world application with students immediately after the training. However, based on the findings of this study, it’s important to recognize that there may be a learning curve in terms of incorporating EQUIP competency-based assessment and techniques beyond initial training sessions. The EQUIP platform offers resources within their Learning Management System (LMS)^1^ to support new users. For instance, participants in this study described anxiety surrounding role-playing, a noteworthy concern for many participants. Although this was briefly covered in the training, folks learning how to use EQUIP can also refer to a dedicated, self-paced learning module on the website, designed to teach faculty, trainers, and supervisors on how to prepare students for this aspect.

Similarly, during the training session on competency-based feedback, the faculty and supervisors were encouraged to refer back to the EQUIP LMS module as needed when applying these techniques in the real-world. Furthermore, the study’s findings seamlessly correspond with the focus on ensuring a competency benchmark when rating. The issue of establishing clear ‘benchmarks’ was covered within the training, emphasizing rater agreement and resolving potential confusion in how to rate competencies, which was particularly pertinent given the varied backgrounds of the participants. Still, it is recognized that more practice using the competency assessment tools and role plays in their work may strengthen the application. As such, it will be integral to do longer term follow-up, both quantitatively and qualitatively, to see long term impacts of the EQUIP training and application of competency-based approaches at Makerere University and Butabika Hospital.

The study’s results resonate with the comprehensive training provided through the EQUIP approach, and as such we recommend those interested in incorporating an EQUIP competency-based approach to follow a similar 15-h training program for preparing faculty, supervisors, and others in the university setting. The carefully designed training modules, addressing concerns such as role-playing anxiety, feedback delivery, and benchmarking, contributed to the participants’ favorable perceptions and experiences. This harmony between training components and research outcomes strengthens the credibility and practicality of the study’s findings, endorsing the potential of the EQUIP approach in clinical psychology education.

The recognition of the significance and utility of role-playing exercises in competency-based training, assessment, and supervision emerges as a notable takeaway. These exercises serve as a dynamic tool for tracking students’ progress and nurturing their confidence in clinical practice. Evidence corroborating the effectiveness of role-playing spans various domains, including medical education and counseling programs [Grant, 2006; Smith, 2009; Sude and Baima, 2020 Jordans et al. (2021a,b)]. It’s noteworthy that occasional student discomfort during role-plays is a shared experience, which can be leveraged for constructive feedback and support. Role-playing offers a simulated platform for students to navigate diverse clinical scenarios, guided by supervisory feedback, thereby identifying areas for growth and honing their skills.

In the realm of competency training and assessment, feedback plays a crucial role that consistently comes to the forefront. Participants consistently emphasize its significance in pinpointing strengths and areas for improvement, serving as a catalyst for self-improvement. The positive effects of encouraging feedback on enhancing self-confidence have been well established by various sources (Nicholls, 2002; Boud, 2007; Watling and Ginsburg, 2019). However, delivering feedback requires a careful approach to prevent defensive student reactions. Supervisors, who embody the roles of mentors, guides, educators, and role models, bear the responsibility of providing feedback on progress and areas needing refinement (Johnson et al., 2007; Johnson, 2015; Tull et al., 2023). Offering constructive feedback empowers students to address their weaknesses while gaining the essential skills, knowledge, and attitudes required to excel as clinical psychologists (Kilminster et al., 2007). The pivotal roles of supervisors as mentors and educators become evident in this context, showcasing their influence on students’ growth and development. By bridging the gap between theory and practice, supervisors empower students to overcome limitations and cultivate the skills necessary for excellence in clinical psychology (Dunne and Rawlins, 2000). This study highlights the vital importance of supervisor involvement in shaping students’ experiences within a competency-based learning framework, thus deepen the pivotal role of mentorship in nurturing adept clinicians.

The innovative methodology employed in this study, involving the immediate application of training knowledge, introduces experiential learning as a potent strategy. This study prompts the necessity for longitudinal research to gauge the enduring impact and sustainability of the acquired competencies. Continuously tracking participants’ progress and utilization of EQUIP resources over time assumes significance to assess the approach’s sustained efficacy, thereby yielding insights into the potential for ongoing support or refresher training. The concept of sustained utilization of EQUIP resources post-training carries noteworthy implications. It signifies a commitment to integrating these resources into regular teaching and assessment practices, potentially heightening student competencies and clinical outcomes over the long haul. This integration demands a sustainable and user-friendly platform, stress-out the importance of fostering an encouraging environment that nurtures ongoing engagement. In conclusion, this study propels our comprehension of competency-based education within the realm of clinical psychology in university settings. Its alignment with prior research, emphasis on role-play and feedback, acknowledgment of supervisor roles, and insights into training duration collectively contribute to the ongoing refinement of clinical psychology education. The study’s implications are profound for educational institutions seeking to embrace competency-based approaches, advocating for comprehensive training and nurturing environments. Furthermore, the study’s pioneering approach and its enduring impact emphasize the demand for continuous research and enhancement in competency-based education strategies.

### Limitation

Possible limitations of this study include a small sample size, reliance on self-report data, and the short follow-up period. These limitations call for caution in generalizing the findings and highlight the need for further research to address these gaps and provide a more comprehensive understanding of the EQUIP approach’s feasibility and effectiveness.

### Key recommendations

Training in EQUIP and Continued Utilization of Learning Resources: Practical, competency-based training in how to use EQUIP resources will support faculty, supervisors, and other university staff in feasibly implementing these techniques. Additionally, continued learning or “refreshers” should be emphasized through access to the EQUIP LMS. These freely accessible modules are self-paced learning and can act as useful and detailed reminders for certain techniques while supporting different learning styles.

Longitudinal Studies: To gauge the long-term impact of the competency-based approach, institutions are encouraged to conduct longitudinal studies collecting both qualitative and quantitative data. These studies can follow participants who have undergone the training and track their application of EQUIP principles over an extended period (e.g., 3, 6, 9, or 12 months). Longitudinal research will provide insights into the sustained effectiveness of the approach in real-world clinical settings.

Collaborative Implementation: Establish platforms for collaborative implementation and continuous improvement within the university and with other universities and institutions applying the same concepts. Regular forums, workshops, and discussion groups can facilitate the exchange of experiences, strategies, and best practices among educators, supervisors, and faculty members. This collaborative approach will foster a supportive community focused on refining the application of the competency-based approach.

Institutional Commitment: Institutional leadership and administration should recognize the significance of competency-based education and allocate resources to support its successful implementation. This includes not only dedicating time and funds for training but also creating an environment that encourages ongoing professional development and the incorporation of innovative teaching methodologies.

## Conclusion

This study offers strong proof of the viability and importance of introducing the EQUIP competency-based approach into clinical psychology students’ pre-service training. Participants—faculty members and supervisors—acknowledged that the strategy might guarantee uniformity in proficiency levels, addressing Uganda’s lack of a defined curriculum. The organized design of the EQUIP tools—especially the ENACT tool—made them useful tools for educating and evaluating students. The participants conveyed their confidence that, with continuous application, a competency-based approach like this may improve the general caliber of clinical psychology training. The use of role-plays in training, assessment, and supervision of competencies was deemed important and beneficial by participants. Although role-playing may cause some students to feel uncomfortable at first, research indicates that it works well in a variety of educational settings, and the EQUIP approach successfully incorporates this style. It has been shown that role-playing activities are quite helpful in tracking students’ development and boosting their self-assurance during clinical practice. The EQUIP approach’s integration of role-plays is in line with accepted teaching methodologies and gives students hands-on experience that mimics real-world scenarios. Feedback emerged as a crucial component in competency training and assessment. Participants acknowledged that feedback, when delivered thoughtfully, empowered them to identify strengths and weaknesses, fostering a commitment to improvement. It was emphasized that supervisors’ dual roles as educators and mentors in giving feedback are crucial for assisting students in their development into qualified clinical psychologists. To sum up, this pilot project makes a substantial contribution to the conversation on competency-based training and assessment in clinical psychology, especially in environments with limited resources like Uganda. The results recommend more assistance and training to guarantee the EQUIP approach’s effectiveness. Including role-plays and providing feedback is essential to optimizing the advantages of this novel training approach, as is resolving related issues. To ensure the development of competent practitioners, the research suggests that future clinical psychology and psychotherapy courses adopt comparable competency-based approaches with an emphasis on hands-on training techniques, standardized role-plays, and strong feedback systems.

## Figures and Tables

**Figure 1.**
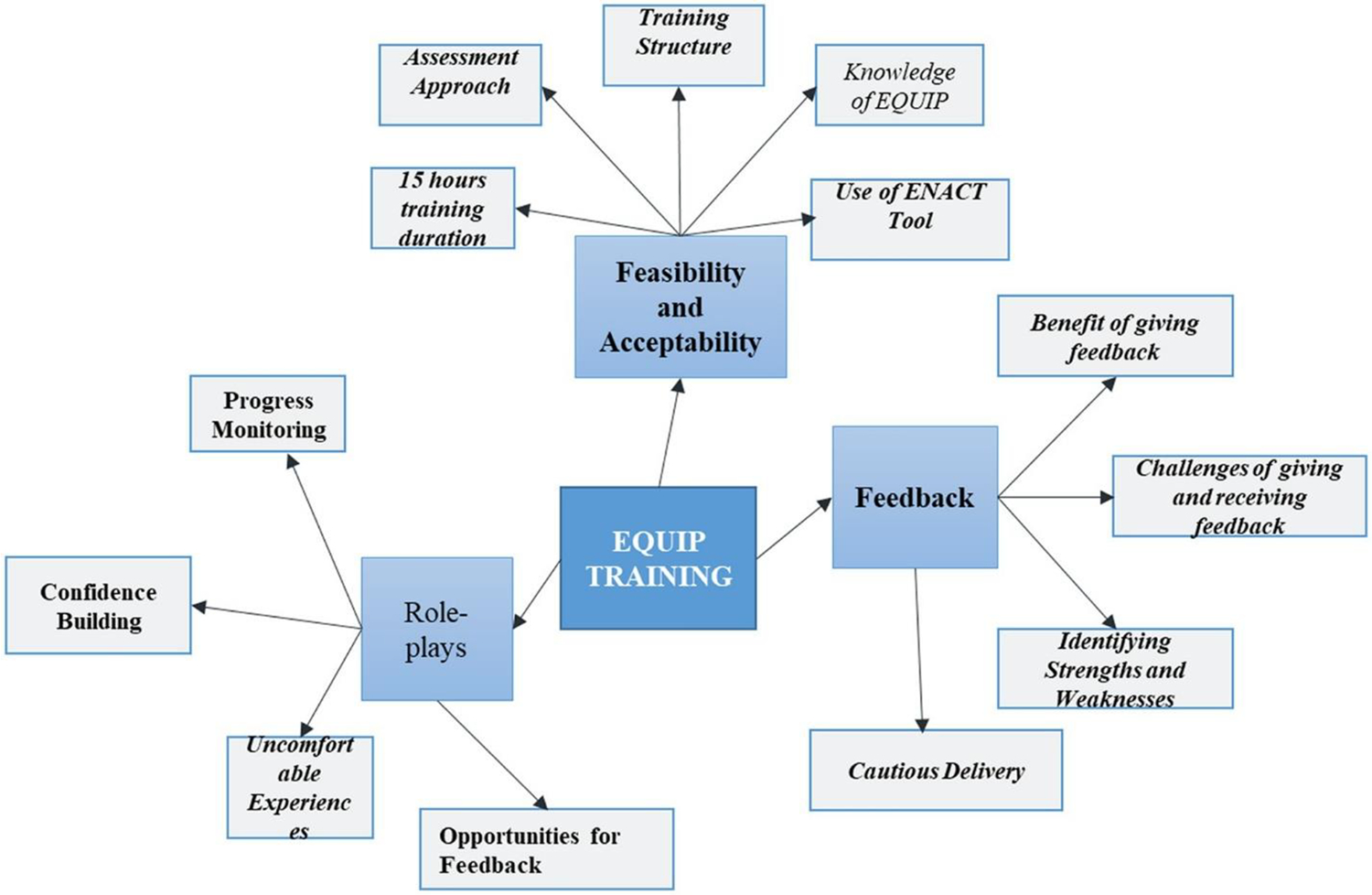
Thematic map.

## Data Availability

The raw data supporting the conclusions of this article will be made available by the authors, without undue reservation.

## Footnotes

1.
^https://equipcompetency.org/en-gb/resources
